# Opioid-Free Anaesthesia Effectiveness in Thoracic Surgery—Objective Measurement with a Skin Conductance Algesimeter: A Randomized Controlled Trial

**DOI:** 10.3390/ijerph192114358

**Published:** 2022-11-02

**Authors:** Dominika Sadowska, Szymon Bialka, Piotr Palaczynski, Damian Czyzewski, Jacek Smereka, Anna Szelka-Urbanczyk, Hanna Misiolek

**Affiliations:** 1 Clinical Department of Internal Medicine, Dermatology and Allergology, Faculty of Medical Sciences in Zabrze, Medical University of Silesia, 40-055 Katowice, Poland; 2 Department of Anaesthesiology and Intensive Care, Faculty of Medical Sciences in Zabrze, Medical University of Silesia, 40-055 Katowice, Poland; 3 Department of Thoracic Surgery, Faculty of Medical Sciences in Zabrze, Medical University of Silesia, 40-055 Katowice, Poland; 4 Department of Emergency Medical Service, Wroclaw Medical University, 50-367 Wroclaw, Poland

**Keywords:** skin conductance algesimeter, opioid-free anaesthesia, video-assisted thoracic surgery, acute pain

## Abstract

Background: Chest surgery is associated with significant pain, and potent opioid medications are the primary medications used for pain relief. Opioid-free anaesthesia (OFA) combined with regional anaesthesia is promoted as an alternative in patients with an opioid contraindication. Methods: Objective: To assess the efficacy of OFA combined with a paravertebral block in pain treatment during video-assisted thoracic surgery. Design: A randomized, open-label study. Setting: A single university hospital between December 2015 and March 2018. Participants: Sixty-six patients scheduled for elective video-assisted thoracic surgery were randomized into two groups. Of these, 16 were subsequently excluded from the analysis. Interventions: OFA combined with a paravertebral block with 0.5% bupivacaine in the OFA group; typical general anaesthesia with opioids in the control group. Main outcome measures: Intraoperative nociceptive intensity measured with a skin conductance algesimeter (SCA) and traditional intraoperative monitoring. Results: Higher mean blood pressure was observed in the control group before induction and during intubation (*p* = 0.0189 and *p* = 0.0095). During chest opening and pleural drainage, higher SCA indications were obtained in the control group (*p* = 0.0036 and *p* = 0.0253), while in the OFA group, the SCA values were higher during intubation (*p* = 0.0325). SCA during surgery showed more stable values in the OFA group. Pearson analysis revealed a positive correlation between the SCA indications and mean blood pressure in both groups. Conclusions: OFA combined with a paravertebral block provides effective nociception control during video-assisted thoracic surgery and can be an alternative for general anaesthesia with opioids. OFA provides a stable nociception response during general anaesthesia, as measured by SCA.

## 1. Introduction

Thoracic surgery is associated with the most severe pain, requiring the use of analgesics at maximal or submaximal doses [1]. For elective VATS (VATS; Video-Assisted Thoracic Surgery), the pain can result from several reasons. One of the most important causes of this pain is damage to the intercostal nerve [2]. During VATS, the incisions for the tubes and the retractions and other manipulations can cause damage to and inflammation of both the intercostal nerve and associated muscles, leading to serious chronic pain following surgery [3]. Furthermore, the expected pain reduction after switching from open thoracotomy to VATS did not occur and the chronic pain prevalence remained similar, comparing these two procedures [4]. The difficulty in controlling pain after thoracic procedures is caused by anatomical reasons, surgical technique, and the underlying disease (cancerous infiltration and trauma). Moreover, patients subjected to thoracic surgery are at particular risk of developing postoperative pulmonary complications resulting from primary respiratory dysfunction and one-lung ventilation [5]. The pain can impede the treatment, extend the hospital stay, increase costs, and reduce the quality of life; therefore, it is necessary to find a safe and effective method of analgesia for thoracic surgery [6].

Currently, the most commonly used analgesics for thoracic pain are opioids [3,6]. However, they are associated with a large range of adverse effects, such as ICU-related delirium, respiratory depression, increased requirements and the duration of mechanical ventilation, and a post-operative ileus or a delay in the recovery of intestinal function [7,8]. The recommended analgesic management for surgical pain is multimodal therapy, based on analgesics with different mechanisms of action. One of the multimodal analgesia methods is opioid-free anaesthesia (OFA; Opioid-Free Anaesthesia). The overall concept is based on replacing opioids during anaesthesia with other analgesics and combining them with regional analgesia to minimize surgical pain [9,10]. Favourable effects of OFA in general, bariatric, and gynaecological surgery have been reported [11,12]. One specific advantage of OFA in VATS is that the incision site pain can be managed without opioids [13]. Thoracic paravertebral blocks are equal to epidural analgesia in the management of VATS-related pain, with fewer adverse events and a higher success rate.

An effective method of measuring pain in anaesthetized patients is important due to the fact that early intraoperative pain management can lead to decreased post-operative and chronic pain [14,15]. One way of measuring intraoperative pain is the use of a pain monitor, a device for analysing certain physiological factors to determine the severity of pain. One of the few commercially available devices is the skin conductance algesimeter (SCA; Skin Conductance Algesimeter), where the effect of pain on the body causes increases nervous activity that can lead to sweating in the hands and the feet. As the sweat glands are activated in this response to pain, the skin’s innate resistance decreases and the electrical conductance of the skin increases, allowing for a specific response per skin measurement (skin conductance responses per second or SCR) [16]. Non-invasively measured analogue data on skin conductivity are transmitted to the central unit, converted to digital data, and analysed, including fluctuations [17,18,19]. Other measurement methods, such as the analgesia nociception index and the surgical pleth index, are influenced by other factors and can be inaccurate [20].

The goal of this study was to determine the safety and effectiveness of OFA and the nociception response using skin conductance fluctuations in patients undergoing video-assisted thoracic surgery.

## 2. Materials and Methods

This randomized, controlled study was conducted at the Medical University of Silesia, Poland, with approval by the institutional review board (approval No.: KNW/0022/KB1/41/16). We enrolled 66 patients scheduled for elective VATS between December 2015 and March 2018. All patients provided explicit written informed consent to participate in the study. The trial was registered at ClinicalTrials.gov (registration No.: NCT04355468). In addition, we confirm that all methods were performed following the relevant guidelines and regulations. The patients were aged 18–65 years, with a body mass index (BMI; Body Mass Index) of 19–30 kg∙m^–2^ and an American Society of Anesthesiologists (ASA; American Society of Anesthesiologists) physical status of I–III. The exclusion criteria included significant coagulopathy, contraindication to thoracic paravertebral block (ThPVB; Thoracic Paravertebral Block) or medication enumerated in the protocol, history of chronic pain, chest wall neoplastic invasion, previous thoracic spine surgery, and renal failure (glomerular filtration rate < 60 mL∙min^–1^∙1.73 m^–2^). Chronic pain was defined as the daily usage of opioids for the management of pain and/or the patient being seen by pain management specialists for chronic pain management.

### 2.1. Protocol

Patients were randomly assigned to 2 groups receiving different intraoperative analgesic regimens:

- Typical general anaesthesia with opioids (control group);
- OFA and ThPVB (OFA group).

Randomization without stratification was based on computer-generated codes, which were kept confidential in sequentially numbered opaque envelopes. All patients were premedicated with oral midazolam in adequate doses.

In the control group, fentanyl (Fentanyl WZF, Polfa Warszawa S.A., Warsaw, Poland) was used for surgical analgesia. For anaesthesia induction, a dose of 1.5 µg∙kg^–1^ was administered; then, fractional doses of 1–3 µg∙kg^–1^ were applied if the heart rate (HR; Heart Rate) or mean blood pressure (MBP; Mean Blood Pressure) increased by more than 20% above the baseline value obtained just before surgery commencement.

In the OFA group, before the induction of general anaesthesia, a single-shot ThPVB was performed at the Th3–Th4 level, approximately 2.5–3 cm laterally to the tip of the spinous process. A pre-block ultrasound examination was performed to assess the depth of the transverse process and the pleura. An insulated 10 cm needle was used, which was connected to a peripheral nerve stimulator with an initial set current of 2.5 mA. The current was gradually reduced as the needle was inserted deeper until the appearance of visible intercostal muscle activity with a current of 0.3–0.5 mA (paravertebral space identification). An amount of 0.5% bupivacaine (0.3 mL∙kg^–1^) was then injected after a negative aspiration test for air or blood. The efficacy of the blockade to cold was checked after 20 min symmetrically on both sides of the thorax with a plastic ampoule of saline kept in a freezer. A difference in the sensation of cold between the blocked and unblocked sides of the thorax was assumed to indicate an effective block. After ThPVB was performed, a continuous intravenous infusion of lidocaine and ketamine was started in the following scheme:

1. Immediately after anaesthesia induction, lidocaine (Lidocaine hydrochloride WZF, Polfa Warszawa S.A., Poland) was administered as an i.v. bolus at a dose of 1.5 mg∙kg^–1^ and ketamine (Ketalar, Pfizer, Poland) in an i.v. bolus of 0.35 mg∙kg^–1^;
2. This was followed by an infusion of lidocaine at 2.0 mg∙kg^–1^∙h^–1^ for 2 h, continued at a dose of 1.2 mg∙kg^–1^∙h^–1^, and ketamine infusion at 0.2 mg∙kg^–1^∙h^–1^ for 2 h, continued at a dose of 0.12 mg∙kg^–1^∙h^–1^.

In both groups, general anaesthesia was induced with midazolam at 0.1 mg∙kg^–1^, propofol at 2 mg∙kg^–1^, and cisatracurium at 0.15 mg∙kg^–1^. The patients were intubated with a left-sided double-lumen tube of an adequate size and positioned laterally. Anaesthesia was maintained with 1 minimum alveolar concentration of sevoflurane. The patients awoke from anaesthesia in a post-anaesthesia care unit and were extubated after the administration of adequate doses of atropine and neostigmine.

### 2.2. Measurements

Demographical (age, sex, height, weight, and BMI) and clinical data (comorbidities and arterial blood pressure) were recorded before surgery. During anaesthesia, the following measurements were performed in all patients: electrocardiography (3-lead), HR (beats∙min^–1^), non-invasive blood pressure (systolic, diastolic, and mean blood pressure) (mmHg), end-expiratory carbon dioxide (mmHg), sevoflurane pressure, and arterial oxygen saturation measured by a finger pulse oximeter.

During general anaesthesia, the skin conductance fluctuations were assessed with an algometer (Med-Storm Pain Monitor, Med-Storm Innovation AS, Oslo, Norway) involving the number of conductance oscillations per second. The measurement was based on the changes in skin conductance that arose due to pain stimulus. During the first 20 min, before general anaesthesia induction and after the placement of SCA electrodes, the parameters of skin conductance were recorded continuously until the stabilization of measurements. Data from SCA and haemodynamic parameters were documented throughout the anaesthesia and subjected to statistical analysis concerning the following events: before anaesthesia induction (B), during intubation (I), chest opening (O), pleural drainage (D), chest closing (C), and after anaesthesia (A).

### 2.3. Statistical Analysis

The data with a normal distribution are presented as the mean ± SD. Non-normally distributed and ordinal data are presented as the median with the upper and lower quartiles, minimum, and maximum. Qualitative data are presented as numbers. The normal distribution was evaluated by the Shapiro–Wilk test. For comparison between the groups, Student’s *t*-test was used for independent variables (homogeneity of variances was tested with Levene’s test) and the Mann–Whitney U test for other data. To compare dichotomous variables, we utilized chi-squared tests with Yates correction. Parametric variance analysis with repetitive measurements and post hoc contrast analysis were performed to analyse the variability of the parameters over time and their between-group differences. An alternative Friedman test was used and supplemented with Dunn–Bonferroni post hoc tests. The degree of correlation of pain monitor indications with HR and MBP was evaluated by calculating the coefficient of determination (Pearson’s correlation coefficient square) and subjected to statistical verification using the Snedecor F test. A *p*-value less than 0.05 was considered statistically significant. *p*-values were corrected with Bonferroni correction for multiple comparisons. Data were analysed with Statistica 13.0. (TIBCO Inc., Palo Alto, CA, USA).

## 3. Results

**Figure 1:**
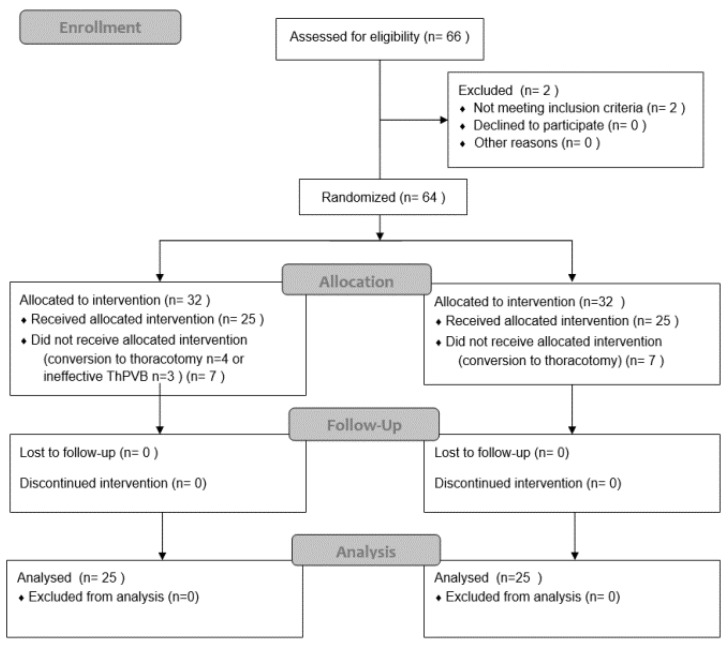
Consort flow diagram.

During the study period, 66 patients who underwent VATS were screened. Based on the inclusion criteria, 64 patients were randomized and assigned to two equal study groups. Overall, 14 patients were excluded after randomization: 7 from the OFA group (4 with conversion to thoracotomy and 3 with ineffective ThPVB) and 7 from the control group (conversion to thoracotomy) (Figure 1). Fifty patients (twenty-one men and twenty-nine women) aged 59 ± 5 years with a BMI of 27 ± 2 kg∙m^–2^ completed the study. Their demographic and clinical characteristics are presented in Table 1. There were no significant differences between groups in age, gender, BMI, or ASA physical status. There was no significant between-group difference in the baseline haemodynamic parameters (Table 1), except for MBP. No significant differences existed between the groups regarding the incidence of comorbidities, except for a higher rate of lung cancer in the study group (Table 2). There were no differences between the groups in the procedures performed (Table 3).

No significant differences between the groups concerning systolic blood pressure (SBP) or HR were noted at the subsequent measuring points. In the control group, we observed significantly higher MBP values before anaesthesia induction and significantly higher diastolic blood pressure (DBP; Diastolic Blood Pressure) and MBP values during intubation. At the same time, higher SpO_2_ values were found in the control group (time points D, C, and A).

The analysis of SCA indications showed no significant between-group differences before anaesthesia induction, during chest closing, or after anaesthesia. Higher SCA measurements were observed in the OFA group during intubation and simultaneously in the control group during chest opening and pleural drainage (Table 4).

**Figure 2:**
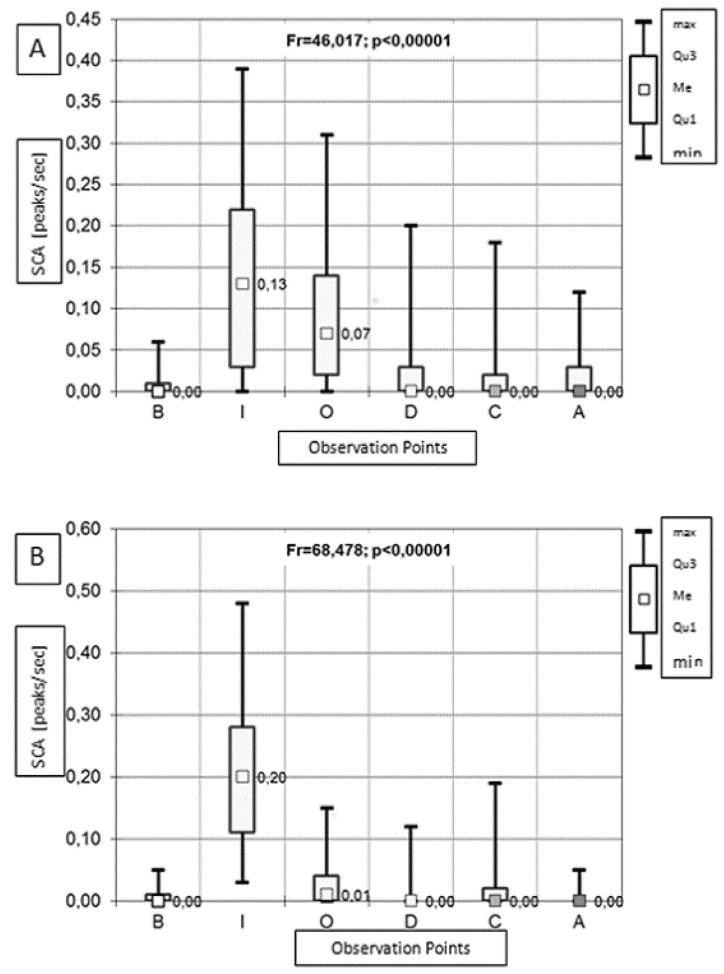
Variability of SCA indications (peaks∙s^–1^) at the consecutive time points in the control group (**A**) and the OFA group (**B**). Data presented as the median (Me), quartile 1 (Qu1), quartile 3 (Qu3), minimum (min)m and maximum (max). SCA, skin conductance algesimeter; OFA, opioid-free anaesthesia; B, before anaesthesia induction; during: I, intubation; O, chest opening; D, pleural drainage; C, chest closing; and A, after anaesthesia.

SCA indication analysis revealed significant variability during anaesthesia in both groups. In the control group, there was a significant increase in the SCA values during intubation and chest opening. Subsequently, a decrease in SCA indications was observed before they returned to the baseline in the final phase of the procedure (time points D, C, and A) (Figure 2A and Table 5). In the OFA group, there was a significant increase in SCA values only during intubation. At time points O, D, C, and A, we observed a significant decrease in SCA indications compared with baseline values (Figure 2B and Table 5).

In the control group, the analysis of the SCA, HR, and MBP values showed statistically significant correlations between SCA and MBP during intubation (R^2^ = 0.2614; *p* = 0.009), chest opening (R^2^ = 0.3381; *p* = 0.0023), and chest closing (R^2^ = 0.2609; *p* = 0.009). In the OFA group, a positive correlation was observed between SCA and MBP only during chest closing (R^2^ = 0.5302; *p* = 0.0004).

## 4. Discussion

Recent studies have shown that analgesia in thoracic surgery is still demanding for medical staff [15]. General anaesthesia excludes using classic pain assessment scales during the procedure, and pain evaluation was mainly based on haemodynamic parameters, i.e., fluctuations in blood pressure and HR. These values are important in monitoring the patient and correlate with the depth of anaesthesia; however, depending on many factors other than pain stimuli limits their use solely as pain-measuring metrics.

A pain monitoring device allows for the objective measurement of pain with reports of effective use in intensive care units. Six et al. presented a case of an 80-year-old patient who, after a neurological trauma with subsequent paresis and aphasia, was subjected to palliative sedation assessed with the Ramsay scale [21]. The NeuroSENSE Monitor and analgesia nociception index monitor were used to evaluate the pain level. Depending on the used scales and devices, there were differences in the assessment of the depth of sedation and degree of pain. As a result, the pharmacological treatment was intensified. Another study compared the usability of the analgesia nociception index monitor and an EEG-based monitor with the visual analogue scale in terminally ill patients. The authors observed cases of insufficient pain management and sedative treatment based on objective pain assessment methods [22]. This discovery led to a modification of pharmacotherapy and clinical decision-making, which increased the patients’ quality of life.

Newborns can also benefit from the use of pain monitors. Passariello et al. examined the effectiveness of sucrose administered orally as a non-pharmacological method of reducing pain in newborns subjected to minor invasive procedures (blood tests) [23]. For pain assessment, they used SCA indications. In 24% of cases, the effectiveness of the method was demonstrated owing to the objective pain measurement. Similarly, a study by Karpe et al. used skin conductance as an objective tool to evaluate pain and discomfort during invasive procedures in the intensive care of neonates, and demonstrated that, despite sedation and analgesia, neonates experienced discomfort related to the therapeutic and diagnostic procedures [24]. Those studies showed that pain monitoring can be useful in patients who cannot communicate efficiently.

In a more related study, Hansen et al. utilized SCA to assess pain during the removal of chest tubes after lung surgery and found that the SCA scores increased during painful procedures [16]. This study shows that SCA can be utilized to measure pain in patients with painful thoracic stimuli and to use it for clinical decision-making. A study by Khanna et al. showed, in critically ill patients who cannot communicate, a similar situation to patients under general anaesthesia—that SCA can be utilized to measure pain and clinical decision-making [25].

Comparing both methods of anaesthesia—the classical one, with the use of opioids, and OFA—we observed that, in three patients from the study group, the paravertebral block was insufficient, requiring the administration of opioids. The result was similar to ThPVB in breast surgery. Paleczny et al. rated this method as technically simple and effective in 75–90% [26] and postulated that the anaesthesiologist’s experience significantly increased the chance of the correct location of the paravertebral space and affected the quality of anaesthesia. The incidence of complications of ThPVB ranges from 1.8 to 10%, with the most dangerous being spinal anaesthesia, pneumothorax, pulmonary haemorrhage, and neurological events [26]. In our study, no clinically significant complications were observed, except for one vessel puncture. There were no significant blood pressure decreases, although they usually accompany sympathetic blockade; this stability could be a beneficial effect of ketamine.

During the surgeries, the cardiovascular parameters were similar in both groups. Significant differences in SCA indications were noted between the groups. Intubation is the most severe stimulus for surgical patients, and dual-lumen tube intubation is a more traumatic procedure and involves a greater risk of complications than classical intubation [27]. Our study confirmed lower SCA values during intubation in the control group, which were related to the beneficial effects of opioids. Differences in the intensity of pain were observed during chest opening and drain insertion. SCA reported significantly lower pain intensity in the study group, which can demonstrate the benefits of ThPVB application. The positive correlation between pain monitoring and MBP values necessitates the use of MBP in intraoperative nociception observation, in contrast to HR, for which there was no positive correlation.

## 5. Conclusions

OFA combined with a paravertebral block provided effective nociception control during video-assisted thoracic surgery and could be an alternative for general anaesthesia with opioids. OFA provided a stable nociception response during general anaesthesia, as measured by SCA.

## 6. Limitations

The small number of patients (mainly from conversion to thoracotomy) was the main limitation. Another one was the small proportion of participants with chronic obstructive pulmonary disease—the greatest benefits resulting from the use of OFA occur in this population, as opioid usage has been proven to increase adverse pulmonary events in adults with COPD [28]. A similar group is obese patients, who were excluded from the analysis.

## Figures and Tables

**Table 1:** Demographic and main patient characteristics.

Variables	OFA Group (n = 25)	Control Group (n = 25)	*p*-Value
Age (years)	60 ± 5	58 ± 6	0.268
Male/female (n)	11/14	10/15	0.572
BMI (kg∙m^–2^)	27.5 ± 4.9	26.9 ± 4.9	0.319
Height (m)	1.69 ± 0.07	1.66 ± 0.08	0.13
Weight (kg)	83 ± 16	81 ± 14	0.60
ASA class, I/II/III (n)	1/15/9	1/14/10	0.49
Surgery time (min)	148 ± 45	145 ± 37	0.68
SBP (mm Hg)	136.5 ± 20.5	142.9 ± 22.1	0.146
DBP (mm Hg)	81.0 ± 10.5	83.8 ± 10.6	0.172
MBP (mm Hg)	102.0 ± 14.2	109.2 ± 14.4	0.043
HR (beats∙min^–1^)	70 ± 11	69 ± 10	0.67

Data presented as the mean ± SD or n. Baseline cardiovascular parameters on entry to the post-anaesthesia care unit. OFA, opioid-free anaesthesia; BMI, body mass index; ASA, American Society of Anesthesiologists; SBP, systolic blood pressure; DBP, diastolic blood pressure; MBP, mean blood pressure; and HR, heart rate.

**Table 2:** Comorbidities in the study groups.

	Group	Yes	No	*p*-Value
Hypertension	Control	16	9	0.387
	OFA	14	11	
Coronary artery disease	Control	7	18	0.500
	OFA	8	17	
Diabetes mellitus	Control	1	24	0.174
	OFA	4	21	
Lung cancer	Control	20	5	0.025
	OFA	25	0	
Nicotinism	Control	5	20	0.371
	OFA	7	18	
Chronic obstructive pulmonary disease	Control	2	23	0.209
	OFA	5	20	
Neurological disorders	Control	1	24	0.305
	OFA	3	22	

Data presented as n. OFA, opioid-free anaesthesia.

**Table 3:** Surgery types in the study groups.

Type of Surgery	OFA Group (n = 25)	Control Group (n = 25)
Lobectomy	13	15
Double lobectomy	6	5
Lung parenchymal resection	6	5

**Table 4:** Skin conductance algesimeter indications (peaks∙s^–1^) at different time points.

Study Phase	Group	Mean	SD	*p*-Value
B	Control	0.010	0.017	0.4225
	OFA	0.010	0.017	
I	Control	0.140	0.122	0.0325
	OFA	0.205	0.125	
O	Control	0.088	0.087	0.0036
	OFA	0.026	0.037	
D	Control	0.029	0.051	0.0253
	OFA	0.009	0.025	
C	Control	0.022	0.047	0.4867
	OFA	0.029	0.057	
A	Control	0.018	0.033	0.1179
	OFA	0.006	0.014	

Data presented as the mean ± SD. OFA, opioid-free anaesthesia; B, before anaesthesia induction; during: I, intubation; O, chest opening; D, pleural drainage; C, chest closing; and A, after anaesthesia.

**Table 5:** Dunn–Bonferroni test results of SCA changes at the consecutive time points in the control and OFA groups (*p*-values).

	Control Group						
OFA group		**B**	**I**	**O**	**D**	**C**	**A**
	B		<0.001	0.004	1.000	1.000	1.000
	I	<0.001		1.000	0.003	<0.001	<0.001
	O	1.000	<0.001		0.049	0.003	0.007
	D	1.000	<0.001	0.441		1.000	1.000
	C	1.000	<0.001	1.000	1.000		1.000
	A	1.000	<0.001	0.309	1.000	1.000	

SCA, skin conductance algesimeter; OFA, opioid-free anaesthesia; B, before anaesthesia induction; during: I, intubation; O, chest opening; D, pleural drainage; C, chest closing; and A, after anaesthesia.

## Data Availability

The datasets used and/or analyzed during the current study available from the corresponding author on request.

## Abbreviations

Abbreviations and Acronyms
ASA	American Society of Anesthesiologists
BMI	Body Mass Index
DBP	Diastolic Blood Pressure
HR	Heart Rate
MBP	Mean Blood Pressure
OFA	Opioid-Free Anaesthesia
SCA	Skin Conductance Algesimeter
ThPVB	Thoracic Paravertebral Block
VATS	Video-Assisted Thoracic Surgery
